# New Combination Regimens vs. Fludarabine, Cytarabine, and Idarubicin in the Treatment of Intermediate- or Low-Risk Nucleophosmin-1-Mutated Acute Myeloid Leukemia: A Retrospective Analysis from 7 Italian Centers

**DOI:** 10.3390/jcm14030700

**Published:** 2025-01-22

**Authors:** Giulia Battaglia, Davide Lazzarotto, Ilaria Tanasi, Carmela Gurrieri, Laura Forlani, Endri Mauro, Francesca Capraro, Giulia Ciotti, Eleonora De Bellis, Chiara Callegari, Luca Tosoni, Matteo Fanin, Gian Luca Morelli, Claudia Simio, Cristina Skert, Michele Gottardi, Francesco Zaja, Eleonora Toffoletti, Daniela Damiani, Renato Fanin, Mario Tiribelli

**Affiliations:** 1 Division of Hematology and BMT, Azienda Sanitaria Universitaria Friuli Centrale, 33100 Udine, Italy; giulia.battaglia@hotmail.com (G.B.); renato.fanin@uniud.it (R.F.); 2 Hematology Unit, Department of Engineering for Innovation Medicine, Azienda Ospedaliera Universitaria Integrata di Verona, 37126 Verona, Italy; 3 Hematology Unit, Azienda Ospedale-Università and University of Padova, 35128 Padua, Italy; 4 Hematology Section, Dipartimento di Medicina Specialistica, Ca’ Foncello Hospital, 31100 Treviso, Italy; 5 Hematology Unit, Azienda Ulss3 Serenissima, Ospedale dell’Angelo, 30174 Venice, Italycristina.skert@aulss3.veneto.it (C.S.); 6 Department of Oncology, UOC Oncohematology, Istituto Oncologico Veneto (IOV) IRCCS, 35128 Padova, Italymichele.gottardi@iov.veneto.it (M.G.); 7 Hematology Unit, Azienda Sanitaria Universitaria Giuliano Isontina, 34148 Trieste, Italyfrancesco.zaja@asugi.sanita.fvg.it (F.Z.); 8 Department of Biomedicine and Prevention, University of Rome Tor Vergata, 00133 Rome, Italy; 9 Department of Medicine, University of Udine, 33100 Udine, Italy

**Keywords:** acute myeloid leukemia, *FLT3*, induction therapy, midostaurin, gemtuzumab ozogamicin

## Abstract

**Background**: Nucleophosmin-1 (*NPM1*) mutation accounts for 30% of acute myeloid leukemia (AML) cases and defines either low- or intermediate-risk AML, depending on *FLT3*-ITD mutation. New combination regimens (NCRs), adding midostaurin and gemtuzumab ozogamicin (GO) to the 3 + 7 scheme, are commonly used, though there are no data that compare NCRs with intensive induction chemotherapy. **Methods**: To evaluate the efficacy and safety of NCRs and FLAI in *NPM1*+ AML, we retrospectively analyzed 125 patients treated with FLAI (*n* = 53) or NCRs (*n* = 72) at seven Italian Centers. **Results**: The median age was 61 years and 51/125 (41%) were *FLT3*-ITD+. The complete remission (CR) rate was 77%, slightly better with NCRs (83% vs. 68%; *p* = 0.054). NCRs yielded a superior median overall survival (OS) (not reached (NR) vs. 27.3 months; *p* = 0.002), though the median event-free survival (EFS) was similar (NR vs. 20.5 months; *p* = 0.07). In low-risk AML, CR was higher in NCRs (94% vs. 72%, *p* = 0.02), as were median OS (NR vs. 41.6 months; *p* = 0.0002) and EFS (NR vs. 17.8 months; *p* = 0.0085). In intermediate-risk AML (*FLT3*-ITD+), there were no differences in CR (60% vs. 71%; *p* = 0.5), OS (*p* = 0.27), or EFS (*p* = 0.86); only allogeneic transplantation improved OS (NR vs. 13.4 months; *p* = 0.005), regardless of induction regimen. The safety profile was similar, except for delayed platelet recovery with FLAI (22 vs. 18 days; *p* = 0.0024) and higher-grade II–IV gastrointestinal toxicity with NCRs (43% vs. 18.8%; *p* = 0.0066). **Conclusions:** Our data suggest the superiority of NCRs over FLAI in low-risk patients, while all outcomes were comparable in intermediate-risk patients, a setting in which only transplants positively impacted on survival.

## 1. Introduction

Nucleophosmin 1 (*NPM1*) is a ubiquitous nucleus–cytoplasmic protein that is involved in many important cellular functions, including the maintenance of genomic stability and response to nucleolar stress. *NPM1* represents the most frequently mutated gene in acute myeloid leukemia (AML), accounting for 30% of AML cases, and traditionally confers a better prognosis, although this depends on the pattern of co-mutated genes in the specific patient [1].

In the 5th edition of the WHO classification of hematolymphoid tumors, *NPM1*-mutated AML is a distinct entity and can be diagnosed irrespective of the percentage of blasts on bone marrow or peripheral blood [2]. According to the ELN 2022 risk stratification, *NPM1*-mutated AML without adverse-risk cytogenetic abnormalities can be classified either as low risk or as intermediate risk, according to the presence of an *FLT3*-ITD mutation [3].

Traditional induction therapies for *NPM1*-mutated AML in fit patients rely on intensive chemotherapy such as the classical 3 + 7 or regimens including additional drugs, such as FLAI (fludarabine, cytarabine, idarubicin). In recent years, new combination regimens (NCRs), adding midostaurin (a first-generation *FLT3* inhibitor) or gemtuzumab ozogamicin (GO, an antibody–toxin conjugate that binds to the CD33 epitope on the cellular surface) to the 3 + 7 scheme, have been approved and are commonly used in clinical practice [4,5]. However, the comparison between these NCRs and intensive induction regimens different from 3 + 7, such as FLAI, has not been well established. Therefore, the current study aims to retrospectively compare the efficacy and safety of NCRs (3 + 7+Midostaurine and 3 + 7+GO) vs. an FLAI regimen in a population of low- or intermediate-risk *NPM1*-mutated AML patients.

## 2. Materials and Methods

This is a retrospective observational study conducted at 7 Italian Hematologic Centers including all consecutive adult patients with intermediate- or low-risk *NPM1*-mutated AML receiving induction chemotherapy with either FLAI (2000 mg/sqm of cytarabine, days 1 to 5; 25 mg/sqm of fludarabine, days 1 to 5; and 12 mg/sqm of idarubicin, days 1-3-5) or NCRs (60 mg/sqm of daunorubicin, days 1 to 3; 200 mg/sqm of cytarabine, days 1 to 7; and either 3 mg/sq of GO, days 1-4-7, or 100 mg of midostaurin, days 8 to 21), outside any clinical trial, between 2010 and 2023. The risk stratification was carried out according to the 2022 edition of the European LeukemiaNet (ELN) recommendations for AML (patients diagnosed before 2022 were reclassified accordingly).

This study was approved by the Institutional Review Board (IRB) of the Department of Medicine at the University of Udine.

Primary endpoints were complete remission (CR) rate and measurable residual disease (MRD) negativity rate after induction, overall survival (OS), event-free survival (EFS), and tolerability. Treatment-related toxicity was evaluated according to the Common Terminology Criteria for Adverse Events (CTCAE), version 4.0.

### 2.1. MRD Analysis

MRD was evaluated with quantitative *NPM1* or *WT1* (for patients without a quantitative *NPM1* analysis). Expression analysis of *WT1* and *NPM1* mutation A (*NPM1*mutA) was performed using RNA extracted from bone marrow (BM) samples at diagnosis, after induction therapy, and before allogeneic stem cell transplantation (HSCT). Mononuclear cells were isolated from BM samples on a Ficoll Hystopaque 1077 (Sigma Aldrich Company, St. Louis, MO, USA) density gradient. After total RNA isolation using the QIAmp RNeasy Mini Kit (Qiagen, Hilden, Germany), cDNA transcription and Real-time Quantitative-Polymerase Chain Reaction (RQ-PCR) were performed in accordance with the Europe Against Cancer (EAC) indications for minimal residual disease (MRD) monitoring [6]. Both gene expressions were assessed using absolute quantitative analyses with commercial plasmid curves for the genes of interest and the Abelson control gene (*ABL*).

The *WT1* expression level was obtained using a CE-IVD kit (Ipsogen *WT1* ProfileQuant Kit, Qiagen, Hilden, Germany) designed on exons 1 and 2, according to the European Leukemia Net Study [7], to avoid *WT1*’s mutation hotspot for primer annealing.

The quantification of transcript levels of *NPM1*mutA was obtained using a double-dye oligonucleotide hydrolysis approach (Ipsogen *NPM1* mut A MutaQuant Kit, Qiagen, Hilden, Germany) with primers and probes spanning *NPM1* exons 11 and 12.

All tests were carried out in duplicate on the ABI PRISM 7500 FAST Real-Time PCR System (Applied Byosystems, Foster City, CA, USA). Replicates with ΔCt greater than 1 were repeated. Samples without almost 1000 copies of *ABL* per replicate were rejected.

The mean number of absolute copies of *WT1* or *NPM1*mutA was normalized with respect to the mean number of absolute *ABL* copies. *WT1* results are expressed as normalized copy number every 10^4^ copies of *ABL* (*WT1* copies/10^4^ *ABL* copies) following the EAC indications [8]. Samples above 250 *WT1* copies/10^4^ *ABL* copies [7] were considered overexpressing *WT1*. The *NPM1*mutA transcript levels are reported as normalized copy number every 10^2^ copies of *ABL* (*NPM1*mut/*ABL*%). *NPM1*mutA positivity was defined as at least one of the two replicates with cycle threshold values equal to or less than 40 [9].

### 2.2. Statistical Analysis

The comparison between baseline characteristics among subgroups was obtained using Fisher’s exact or Chi-squared test for categorical variables, Student’s *t*-test for normally distributed variables, and the Mann–Whitney test for non-normally distributed variables. The median follow-up time was calculated among survivors and was last updated in February 2024.

CR was defined according to ELN recommendations. Death during induction was defined as a death occurring within 30 days from the start of the induction therapy. OS was calculated from the date of diagnosis to the date of the last follow-up or to the date of death by any cause. EFS was calculated from the date of diagnosis to the date of the last follow-up, relapse, progression, or death by any cause. OS and EFS were estimated according to the Kaplan–Meier method and the differences between groups were compared with the log rank test. Univariate and multivariate analyses were carried out by Cox regression for OS and EFS.

## 3. Results

### 3.1. Patients

One hundred and twenty-five patients with *NPM1*-mutated AML, treated either with the FLAI regimen (53/125 patients, 42%) or with NCRs (72/125 patients, 58%), were included. The median age was 61 years and 55% of patients were older than 60 years, with no differences in baseline characteristics in the two therapy groups, except for extramedullary involvement (Table 1). All patients had either a normal karyotype or, in a small minority of cases (8/125, 6%), cytogenetic abnormalities that were all “non-high risk”. Globally, 57/125 patients (46%) were *FLT3*-ITD-mutated, while 68/125 (54%) were *FLT3*-wild-type (wt). Among *FLT3*-ITD+ patients, 20 received FLAI and 37 NCRs, while in *FLT3*-wt, 33 were treated with FLAI and 25 were treated with NCRs (*p =* 0.18). Hyperleukocytosis (defined as blast count >30,000/microliter at onset) occurred in 60/125 patients (48%), without differences in the two groups. According to the ELN 2022 recommendations (3), 65/125 patients (52%) were considered at low risk, as well as 32/53 in the FLAI group (60%) and 33/72 in the NCR group (46%) (*p* = 0.15), while 57/125 patients (46%) had an intermediate risk, as well as 20/53 in the FLAI group (38%) and 37/72 in the NCR group (51%) (*p* = 0.15). In three patients, it was not possible to define the risk at diagnosis. The median follow-up was 30 months (range 1–140).

### 3.2. Outcomes and Survival Analysis

Globally, the CR rate after induction was 77% (96/125), with a trend in favor of NCRs (83% vs. 68% in the FLAI group; *p* = 0.054). There were no differences in MRD-negativity rate after induction in the two groups (68% with FLAI vs. 55% with NCRs, *p* = 0.27), nor in death rate during induction (1.9% with FLAI vs. 1.4% with NCRS, *p* = 1.0).

**Figure 1:**
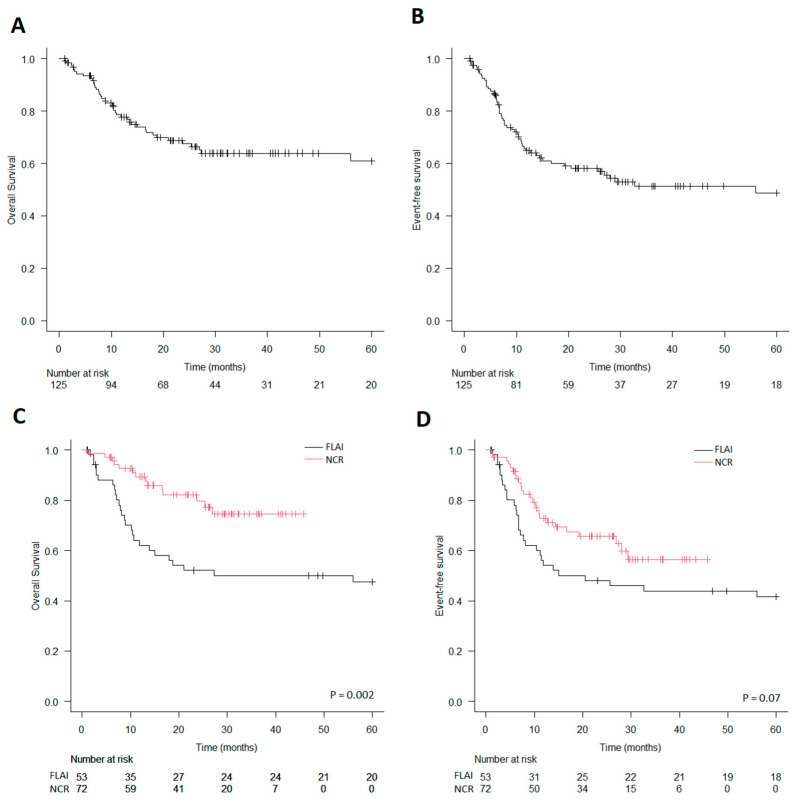
Survival in the whole population. (**A**) Global overall survival. (**B**) Global event-free survival. (**C**) Overall survival stratified by induction therapy. (**D**) Event-free survival stratified by induction therapy.

In the whole population, the 3-year OS and EFS were 64% and 51%, respectively. NCRs performed better than FLAI in terms of OS: the 3-year OS was 75% vs. 50% (*p* = 0.002), while the 3-year EFS was similar (56% vs. 44%; *p* = 0.07) (Figure 1). A multivariate analysis including the following variables—*FLT*3-ITD, hyperleukocytosis, HSCT (as a time-dependent variable), MRD negativity after induction, and type of induction regimen—showed that induction with NCRs and the achievement of MRD negativity were both significant in predicting a superior OS (HR = 0.35, 95% Cl: 0.14–0.85, *p* = 0.034 and HR = 2.57, 95% Cl: 1.07–6.17, *p* = 0.021, respectively), but for EFS, only MRD negativity was significant (HR = 2.27, 95% Cl: 1.15–4.45, *p* = 0.017) (Table 2).

**Figure 2:**
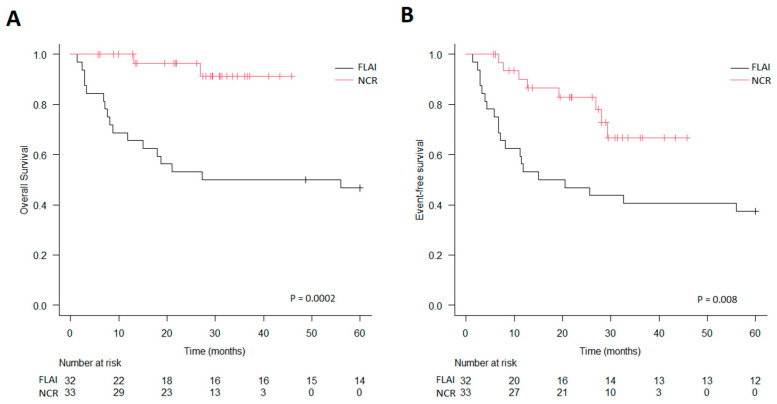
Survival in low-risk patients stratified by induction therapy. (**A**) Overall survival. (**B**) Event-free survival.

In the low-risk group (i.e., *FLT3* wt), the CR rate was significantly higher in the NCR group (94% vs. 72%, *p* = 0.02), and so were both OS (3-year OS of 91% vs. 50%; *p* = 0.0002) and EFS (3-year EFS of 67% vs. 41%; *p* = 0.0085) (Figure 2). The rate of MRD negativity in patients achieving CR was comparable (61% in the FLAI group vs. 72% in the NCR group, *p* = 0.56), and OS and EFS stratified for MRD were not significantly different, though EFS was worse in MRD-positive patients at the end of induction (3-year OS of 77% for MRD-negative patients vs. 69% for MRD-positive patients, *p* = 0.49; 3-year EFS of 62% vs. 30%, *p* = 0.16). In this group, 20/65 patients (30%) underwent HSCT, as well as 11 in the FLAI group and 9 in the NCR group; 14/20 (70%) patients received a transplant for MRD positivity after first-line therapy, while 6/20 (30%) received the same after second-line treatment. In a multivariate analysis including hyperleukocytosis, MRD negativity after induction, HSCT (as a time-dependent variable), and type of induction regimen, only induction with NCRs significantly improved OS (HR = 0.13, 95% Cl: 0.02–0.68, *p* = 0.015).

**Figure 3:**
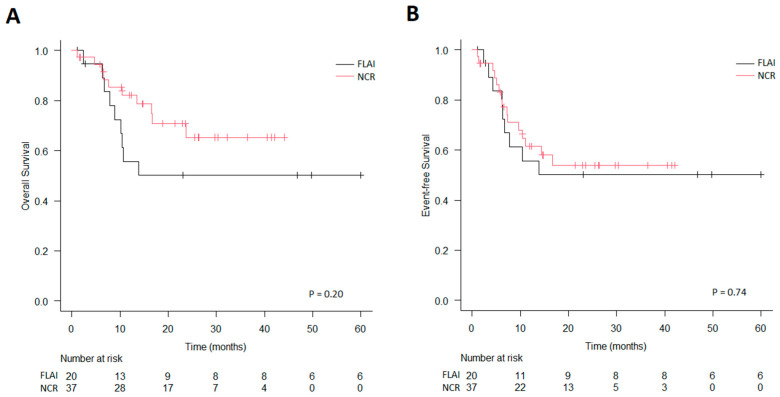
Survival in intermediate-risk patients stratified by induction therapy. (**A**) Overall survival. (**B**) Event-free survival.

In the intermediate-risk group (i.e., *FLT3*-ITD-mutated), there was no difference between the two cohorts in terms of CR (FLAI of 60% vs. NCRs of 73%; *p* = 0.27), MRD-negativity rate in patients obtaining CR (FLAI of 58% vs. NCRs of 44%, *p* = 0.50), OS (3-year OS for FLAI of 50% vs. NCRs of 65%, *p* = 0.20), or EFS (3-year EFS for FLAI of 50% vs. NCRs of 54%, *p* = 0.74) (Figure 3). In this group, 33/57 (58%) patients underwent HSCT, as well as 12 in the FLAI group and 21 in the NCR group; 31/33 (94%) patients received a transplant in the first line and 2/33 (6%) received the same after salvage therapy. In univariate analysis, MRD-negativity was associated with better OS (3-year OS of 80% for MRD-negative patients vs. 50% for MRD-positive patients, *p* = 0.0416) and EFS (3-year EFS of 70% vs. 41%, respectively, *p* = 0.046), and transplanted patients showed improved survival (3-year OS of 77% vs. 32% in patients not transplanted, *p* = 0.0015). However, in multivariate analysis including HSCT (as a time-dependent variable), type of induction, MRD negativity, and hyperleukocytosis, no parameter was significant, though MRD negativity was associated with longer survival (HR = 3.55, 95% Cl: 0.89–14.12, *p* = 0.072); HSCT was not significant (HR = 0.51, 95% Cl: 0.13–2.05, *p* = 0.34).

### 3.3. Safety

Regarding hematologic toxicity, the median recovery time was identical for neutrophils (21 vs. 21 days, *p* = 0.17), while it was longer in the FLAI group for platelets (22 vs. 18 days; *p* = 0.0024). The incidence of febrile neutropenia (FN) and sepsis was similar in the two groups, but the rate of pneumonia was significantly higher in the FLAI group (36% vs. 15% with NCRs, *p* = 0.0107).

The rate of cardiac complications, mostly arrythmias, was not different in the two groups, while the rate of gastro-intestinal toxicity grade ≥2 was higher in the NCR group (43% vs. 19% with FLAI; *p* = 0.0066). The most frequent gastrointestinal complications were mucositis and enteritis.

## 4. Discussion

In the last decade, there have been significant improvements in the knowledge of AML, mainly from a biological point of view, with the discovery of new driving mutations and therapeutic targets. For *NPM1*-mutated AML, new target therapies have emerged and have been combined with the 3 + 7 regimen to improve the outcome. However, data on the comparison of these NCRs with other induction chemotherapy regimens, such as FLAI, are scarce. In this retrospective observational study, we compared the efficacy and safety of NCRs (3 + 7+midostaurin and 3 + 7+GO) vs. the FLAI regimen in a population of low- or intermediate-risk *NPM1*-mutated patients.

Taken together, we observed a survival benefit for NCRs over the FLAI regimen, in line with the literature findings regarding midostaurin and GO, that led to the approval of the two drugs in Europe [4,5]. We acknowledge that the outcome of AML patients is also linked to a consolidation strategy that, in our multicenter study, was highly heterogeneous, though its analysis goes beyond the scope of this work, whose primary objective was to analyze the induction therapy. The survival benefit is evident for OS though not for EFS, and this could be explained by the higher infectious morbidity, mostly pneumonias, observed in the FLAI group. The incidence of pneumonia in our study among patients receiving FLAI (36%) is consistent with data from the literature on similar chemotherapy regimens. Santoni et al. reported a 22.6% incidence of lower respiratory tract infections following induction with FLAI/FLAIE (with Etoposide) [10], while a retrospective cohort study at The University of Texas MD Anderson Cancer Center observed a 19% rate of pneumonia following induction with a chemotherapy regimen that included doses of cytarabine comparable to those used in the FLAI regimen [11].

In the low-risk group, OS and EFS were significantly better in the NCR group. Our data are consistent with the finding that GO, in combination with intensive chemotherapy, is associated with a better response in *NPM1*-mutated AML [12,13]. MRD did not impact on OS or EFS, despite a shorter EFS in MRD-positive patients (with the limits of the small sample size), but considering that HSCT did not impact on OS or EFS, this supports the conclusion that HSCT acts as salvage therapy for MRD-positive or relapsed patients.

In the intermediate-risk group (i.e., *FLT3*-mutated), we observed a similar OS and EFS in the two groups of patients, outlining the good performance of FLAI in *FLT3*-mutated AML, as reported by other studies. For example, Minetto et al. reported that *FLT3* mutational status did not significantly impact OS in patients with *NPM1*-mutated AML treated with the FLAI regimen as the induction [14]. Survival was better only in MRD-negative and HSCT patients, as expected considering the results of the GIMEMA AML1310 trial, where for intermediate-risk MRD-negative patients, an allogeneic transplant was deemed not necessary, as opposed to MRD-positive cases [15]. However, if we consider transplantation as a time-dependent variable in a multivariate analysis, its role is inferior, and this likely depends on the short follow-up of non-transplanted patients, with probably a considerable group of patients who could not reach transplantation, because of early disease progression.

The hematologic toxicities of NCRs were comparable to those of FLAI, except for platelet recovery, which was significantly delayed in the FLAI group, in contrast with the data showing persistent severe thrombocytopenia following GO administration [16]. No patients experienced veno-occlusive disease (VOD) in our cohort. Even though we did not collect data regarding QT prolongation, the rate of cardiac complications was comparable between NCRs and FLAI cohorts, underlining the safety profile of midostaurin concerning cardiac toxicities, also shown in the data from the expanded access program, where QTc prolongation was never associated with clinically significant events [17]. However, gastrointestinal toxicities in the NCR group are of note. Concerning midostaurin, in the RATIFY trial, the percentage of patients showing diarrhea was only 16% [4], but in the expanded access program, it reached 43% [17], in line with the results of our real-life study. GO has also been shown to have gastrointestinal side-effects, with up to a 60% incidence of vomiting and 33% incidence of diarrhea and abdominal pain. Specifically, Grade 3–4 vomiting occurred in 33% and grade 3/4 diarrhea occurred in 14% of cases, as reported in monotherapy studies and post-marketing surveillance [18].

Regarding infections, our study demonstrated an overall incidence of 29% for sepsis and 51% for FN, with no significant differences between the two groups. These rates are slightly more favorable than those reported in the literature; for instance, Santoni et al. observed a 41% incidence of FN and 48% incidence of microbiologically documented infections [10]. Additionally, regulatory studies on midostaurin reported 82% for FN and 52% for sepsis [4], while the GO study reported a 46% incidence of grade 3-4 infections during induction [5]. The FLAI group displayed a greater incidence of pneumonia, possibly due to the more pronounced immunosuppression caused by the fludarabine use.

## 5. Conclusions

In conclusion, in our real-life study, we observed that NCRs perform better than FLAI induction in low-risk patients (*FLT3* wt) in terms of CR rate, OS, and EFS, thanks to the addition of GO. Revisiting the combination of FLAI and GO is of interest, as reported in recent studies [19,20], and could represent another standard of induction chemotherapy when approved by regulatory agencies. Conversely, in the intermediate-risk patients (*FLT3*-ITD-mutated), the results of NCRs were like FLAI in terms of CR rate and survival. In this group, a timely referral to HSCT is of paramount importance, especially in patients with AML persistence after induction [21,22]. Lastly, the issue of NCR safety, mostly in terms of increased gastrointestinal toxicity, should be further investigated.

## Figures and Tables

**Table 1:** Patients’ characteristics at AML diagnosis.

	All Patients	FLAI	NCR	*p*
No. patients	125	53 (42%)	72 (58%)	
Median age (range)	61 (20.0–77.8)	62.2 (26.8–77.8)	59.8 (20.0–74.1)	0.11
Patients > 60 years	69/125 (55%)	33/53 (62%)	36/72 (50%)	0.20
*FLT3*-ITD	57/125 (46%)	20/53 (38%)	37/72 (51%)	0.15
Extramedullary disease	10/125 (8%)	9/53 (17%)	1/72 (1%)	0.002
Hyperleukocytosis	60/125 (48%)	25/53 (47%)	35/72 (48%)	1.00
Low-risk patients	65/125 (52%)	32/53 (60%)	33/72 (46%)	0.15
Intermediate-risk patients	57/125 (46%)	20/53 (38%)	37/72 (51%)	0.15

**Table 2:** Multivariate analysis for OS in general population.

	OS				EFS			
	Hazard Ratio	Lower 95%CI	Upper 95%CI	*p* Value	Hazard Ratio	Lower 95%CI	Upper 95%CI	*p* Value
HSCT	0.5078	0.1650	1.5630	0.23740	0.6609	0.2649	1.649	0.37460
MRD	2.5720	1.0720	6.1720	0.03446	2.2700	1.1570	4.452	0.01705
Type of regimen	0.3525	0.1451	0.8566	0.02135	0.7030	0.3548	1.393	0.31240
Hyperleukocytosis	0.8119	0.3227	2.0420	0.65790	0.8856	0.4370	1.795	0.73600
*FLT3*-ITD	2.0290	0.7616	5.4040	0.15700	1.3460	0.6416	2.822	0.43210

OS: overall survival, EFS: event-free survival, HSCT: hematopoietic stem cell transplant, MRD: minimal residual disease.

## Data Availability

Data and materials are available from the corresponding author upon reasonable request.
